# The dyadic care experiences of elderly individuals with disabilities and caregivers in the home setting from the perspective of family resilience: A qualitative study

**DOI:** 10.3389/fpsyt.2022.963101

**Published:** 2022-10-13

**Authors:** Bahaerguli Abulaiti, Xiangchun Zhang, Tingyu Guan, Meng Wang, Shoumei Jia, Anni Wang

**Affiliations:** School of Nursing, Fudan University, Shanghai, China

**Keywords:** family resilience, elderly individuals with disabilities, caregivers, dyadic coping, care experience, qualitative research

## Abstract

**Background:**

China is one of the most rapidly aging countries in Asia, and nearly 90% of elderly individuals with disabilities choose to receive traditional, family-based, long-term care. A majority of family caregivers have insufficient care capacity and experience physical and emotional distress, which in turn affects the elderly.

**Objective:**

To describe the dyadic care experiences of elderly individuals with disabilities and their caregivers from the perspective of family resilience.

**Methods:**

A phenomenological research method was used. Semi-structured, in-depth, face-to-face interviews with 9 dyads of elderly people with disabilities and their families were conducted from August 2020 to February 2021. The Colaizzi method was used to analyze, summarize, and refine the interview data.

**Results:**

The dyadic care experiences of elderly individuals with disabilities and their caregivers can be summarized in terms of two themes. Theme 1 is dyadic pressure, which includes the following subthemes: (1) substantial objective burden; (2) dual negative experiences, i.e., the perceived low value of elderly individuals and low positive gains by caregivers; and (3) dyadic emotional transmission. Theme 2 is dyadic cooperative coping, which includes the following subthemes: (1) adjustment of family beliefs, including by giving meaning to stress, maintaining a positive outlook toward the future and ensuring spiritual sustentation; (2) changes in family patterns, including flexible adjustment of family patterns and multichannel utilization of social resources; and (3) improvement of family communication, including effective information transmission, rational emotional expression and emotional connection, and cooperation to solve and prevent problems.

**Conclusion:**

Elderly individuals with disabilities and their family caregivers face multiple physical, psychological, and social difficulties and demands during daily care, in which context negative experiences exert influence on this dyad. They collaborate to exhibit family resilience *via* the adaptation and improvement of family beliefs, family patterns and family communication. The family as a unit expends a great deal of effort to adapt to conditions of stress in the context of caring and shows family resilience, which is closely related to the family's cultural background and community situation. Dyadic resilience-based interventions can be developed based on core elements found by this study.

## Introduction

China is one of the most rapidly aging countries in Asia, and it is predicted that by the year 2030, China will fall into an aging problem, with 18.21% of the population over the age of 65, which is far more severe than that of India of 8.45% or Vietnam of 9.45% (1, 2). In 2019, China contained 175.99 million people aged above 60 years old, and by 2030, the population of elderly individuals in China will reach 371 million, accounting for 25.3% of the total population of the country (http://www.cmw-gov.cn/news.view-794-1.html). Disability refers to the loss of physical functions due to chronic diseases, mental disorders or other factors, leading to functional impairment, limited daily activities and difficulties in self-care (3). The increase of life expectancy is often accompanied by the decline of self-care ability and population aging often coexists with disability (4). The disability rate of elderly individuals in China ranges between 10.48 and 13.31% (3), and this figure is projected to rise to 91.62 million by 2030 (3). Among the disabilities in elderly individuals in China, the most prevalent disabilities were hearing loss of 8.3%, physical disability of 6.1%, visual disability of 4.6%. Increasing disability prevalence with age reflects an accumulation of health risks, including chronic illness and injuries (5).

Elderly people with disabilities require emotional, physical, or practical support from family members, relatives, and society until the end of their lives. The old people's mortality increased with increasing disability level (6), and the risk for developing depressive symptoms increased with decreasing ability (7). In the context of traditional cultures and the emphasis on providing for older individuals in Asian countries, most of these people choose to receive family-based long-term care (3). China is a developing country where the pension system does not cover all people, and many elderly people still rely on support from their children. Long-term care for most old people is mainly undertaken by family members in China as well as many Southeast Asian countries (8, 9). In East Asia, especially in China, where collectivism and Confucianism have dominated for more than 2,000 years, living with descendants is a tradition; thus, having descendants to take care of people when they get old is a symbol of happiness. Accordingly, the task of caring for the stress of these families, including both caregivers and elderly individuals with disabilities, is an important issue. Research has shown that family caregivers experience higher levels of stress, deteriorations in their mental and physical health, and disruptions in their social and family relationships (3). Previous studies have found that some caregivers suffer from clinical depression or anxiety, while others experience substantial psychological symptoms (10). When the care burden on these caregivers cannot be addressed effectively, the stress resulting from the task of caring for elderly individuals with disabilities can adversely affect the quality of care and ultimately impact the rehabilitation and prognosis of those elderly individuals (10). However, despite facing many difficulties, some caregivers experience high levels of satisfaction with their caregiving role and thus positive health outcomes. Previous research has found that family function plays an important role in helping caregivers and elderly individuals adapt to this stress. Positive family function contributes to the living status and health status of family members (10). Elderly individuals with better family function exhibit more satisfaction with their care as well as decreased tension (11). The adjustment outcomes can be either negative or positive, and caregivers may derive positive benefits from caregiving, which can act as protective factors for the care burden (12, 13). Excavating strength and potential from a positive perspective has been a concern of an increasing number of researchers (14).

Family resilience theory may provide an integrated perspective that can facilitate adaptation to caregiving stress by the whole family. Family functioning is maintained in the presence of stress *via* the mobilization of resources that are inherent in family resilience (15). Walsh's theoretical model of family resilience, including family belief systems, patterns, and communication, builds on extensive family systems research on transactional processes in well-functioning families, with a primary focus on efficient family functioning in response to adverse conditions, and these key processes of resilience are synergistic (16). Family resilience refers to a process in which the family exhibits functions of coping and adaptation, and it represents a positive process of endurance, self-adjustment and growth that allows families to cope with crisis and changes (10). Studies have shown that enhancing family resilience can lead to advantages for the family and promote family recovery, thereby allowing the family to cope with various difficulties and pressures so that both caregivers and the elderly can achieve better adaptation results (17, 18).

Treating elderly individuals and caregivers as a community that promotes the positive physical and mental adjustment of everyone involved can enhance the life experiences of both parties (10). Current research focuses on elderly individuals with disabilities and their caregivers and ignores the internal interactions, overall features and potential advantages of the family as a whole (10). Therefore, the purpose of this study is to explore the dyadic experience of elderly individuals with disabilities and their caregivers, which can improve our understanding of ways of promoting family adaptation, establish a foundation for future psychosocial support interventions, and benefit other Asian countries in which older people exhibit similar choices of family-based, long-term care.

## Methods

### Design

A descriptive phenomenological study was conducted to describe and explore the care experiences of elderly individuals with disabilities and their family caregivers. The study was reported in accordance with the Comprehensive Standard Guide for Qualitative Research Reports (19). The participants were selected *via* convenience and purposive sampling and were drawn from four communities and two hospitals. The eligibility requirements were as follows. The disabled elderly were: (1) the residents aged 60 or older who were evaluated as mild or more severe on the daily life ability scale (20), (2) primary recipients of home care, (3) no mental disease or cognitive impairment, and (4) voluntarily participated in the research. The “family” in this study refers to the extended family, because aged people often not only live with their spouses, but also with next generations of child-in-law or grandchildren. Primary caregivers, therefore, in this study were (1) aged above 18 years old, (2) self-identified as being primarily responsible for caring for elderly individuals with disabilities in the extended or nuclear family, (3) taking care for more than 1 month, and (4) with no mental disease or cognitive impairment, and voluntarily participated in the research. The daily life ability scale (ADL) is a 10-item scale, including eating, dressing, washing, bowel control, urination control, toileting, walking on level ground, bed and chair transfer, going up and down stairs, and bathing. The total score of 10 items ranged from 0 to 100, and is divided into three levels, with 61–100 as mildly dependent, 41–60 as moderately dependent, and 0–40 as severely dependent. Those paid caregivers were excluded. The sample size was determined by data saturation. Ultimately, nine dyads including elderly individuals with disabilities and their family caregivers participated in the study. The mean age of the elderly individuals with disabilities was 78.5 ± 9.3 years, and the mean age of the family caregivers was 55.3 ± 13.2 years. More details are shown in Table 1.

**Table 1:** General information of the disabled elderly and caregiver.

**ID**	**Elder**										**Carer**									
	**Age (year)**	**Gender**	**Diseases**	**Educational level**	**ADL**	**Religion**	**Disabled length (year)**	**Living situation**	**Income level per person/month (¥)**	**Marital status**	**Age (year)**	**Gender**	**Relation**	**Educational level**	**Religion**	**Marital status**	**Care length (year)**	**Place of residence**	**Sick or not**	**Care length/day (hour)**
1	73	Male	1	Bachelor	80	No	2	With children	3,000–4,999	Divorce	48	Male	Son	Bachelor	No	Married	2	City	No	8–12 h
2	92	Female	2	Illiteracy	50	No	20	With children	1,000–2,999	Widowed	56	Male	Son	High school	No	Married	20	Rural	No	12–24 h
3	81	Male	1	Middle school	0	No	2	With children	1,000–2,999	Widowed	53	Male	Son	High school	No	Married	2	City	No	12–24 h
4	84	Female	3	Illiteracy	85	Buddha	17	With children	<1,000	Unmarried	25	Female	Grand daughter	Middle school	No	Married	17	Town	No	4–8 h
5	82	Female	2	Illiteracy	65	No	25	With spouse	1,000–2,999	Married	59	Female	Daughter	Primary	No	Married	25	Town	No	8–12 h
6	65	Female	2	Middle school	55	No	3	With spouse	1,000–2,999	Married	62	Male	Partner	Middle school	No	Married	3	Town	No	12–24 h
7	82	Male	2	Primary	45	No	5	With spouse	<1,000	Married	78	Female	Partner	Illiteracy	No	Married	5	Rural	Yes	8–12 h
8	62	Male	2	Middle school	70	No	17	With spouse and children	<1,000	Married	58	Female	Partner	Primary	No	Married	17	Town	Yes	8–12 h
9	86	Female	2	Illiteracy	25	No	27	With children	<1,000	Widowed	59	Female	mother -in-law	Middle school	No	Married	27	Town	Yes	8–12 h

E, Elder; C, Caregiver; Hereinafter referred to as C,E.

### Data collection

Data were mainly collected *via* in-depth, face-to-face interviews conducted over a 3-week duration in April 2021. According to the family resilience framework and the relevant literature, the research team employed a semi-structured interview guide. Example questions included the following: How is your physical health? How do you perceive your pressure and difficulties, and how can you solve them? What services do your family and community provide in your life? Probing questions such as the following were also used to elicit additional information and details, e.g., what were the primary factors impacting your experiences? Two researchers conducted this interview in Chinese; one researcher was the main interviewer who asked questions and the other was an assistant. Both researchers are PhD candidates in nursing, who have been trained in qualitative methodology and received the guidance of the correspondence authors. We conducted separate interviews with the aged and caregiver in a quiet room separately at their home where not to be disturbed by others, which ultimately facilitated their free expression. Because Chinese people are often shy to express the feeling in the presence of their family members. Interviews were collected by field recording with notes and took ~1–1.5 h per pair to conduct.

### Data analysis and rigor

We employed the Colaizzi method to analyze, summarize, and refine the interview data, and we used NVivo 11.0 software to code and store the data. This study is to understand the dual daily care experience of the disabled elderly at home and their caregivers, such that how the care pressure is experienced. The Colaizzi method is, therefore, suitable with focusing on interpreting the feelings of the respondents, and involves a step of confirmation results back to the participants.

Multiple strategies were used to establish qualitative rigor (21). To enhance credibility, we engaged other team members so that non-coders could review and challenge our interpretations. All interviews were conducted using semi-structured interview guidelines to ensure the rigor of the data collection process. All interviews were conducted by the two interviewers (ZXC and GTY). These interviews had a long duration to ensure authenticity and data saturation. Participants' statements were clarified during interviews, and data were recorded, transcribed verbatim, cross-checked, and returned to participants for correction. Interviews and data analysis were translated from Chinese to English by a bilingual investigator and reviewed by a second bilingual investigator to verify consensus and thereby enhance reliability. Specifically, each transcript was encoded independently by two investigators after cross-checking and discussion of the codes and topics; these two values were combined to determine the final transcript code. The codes were then grouped in accordance with similarities and differences, and the semantic content of the code was validated further. The first and corresponding authors worked together to adjust the topic to address the entire dataset. Finally, the first author translated the topic and quotations into English, while the corresponding author verified the Chinese-English comparison sentence by sentence. To further ensure verifiability, we held frequent coding team meetings to discuss code usage, coding, refined definitions, and the translation, and any disagreements were resolved by the full group. We focused on the perceptions and experiences of these dyads, compared overlaps and contrasts, and enhanced our understanding of the dyads' relationships and experiences to ensure that the explanations and assumptions resulting from the analysis were sound. In qualitative research, rich descriptions of duality and participant quotations are considered to contribute to transference. Verifiability is achieved if credibility, transferability, and auditability are established (22).

### Ethical approval

This study was approved by the Institutional Review Board of Research Institutions (IRB). Participation was entirely voluntary, verbal and written consent were obtained prior to all interviews, and the informants' anonymity and right to withdraw from the study prior to analysis were guaranteed. The interviews were conducted when the participants felt alert and ready. Contact information for psychological assistance was provided if needed. All participant information collected in the context of this study was anonymous and stored securely by the researchers.

## Results

**Figure 1:**
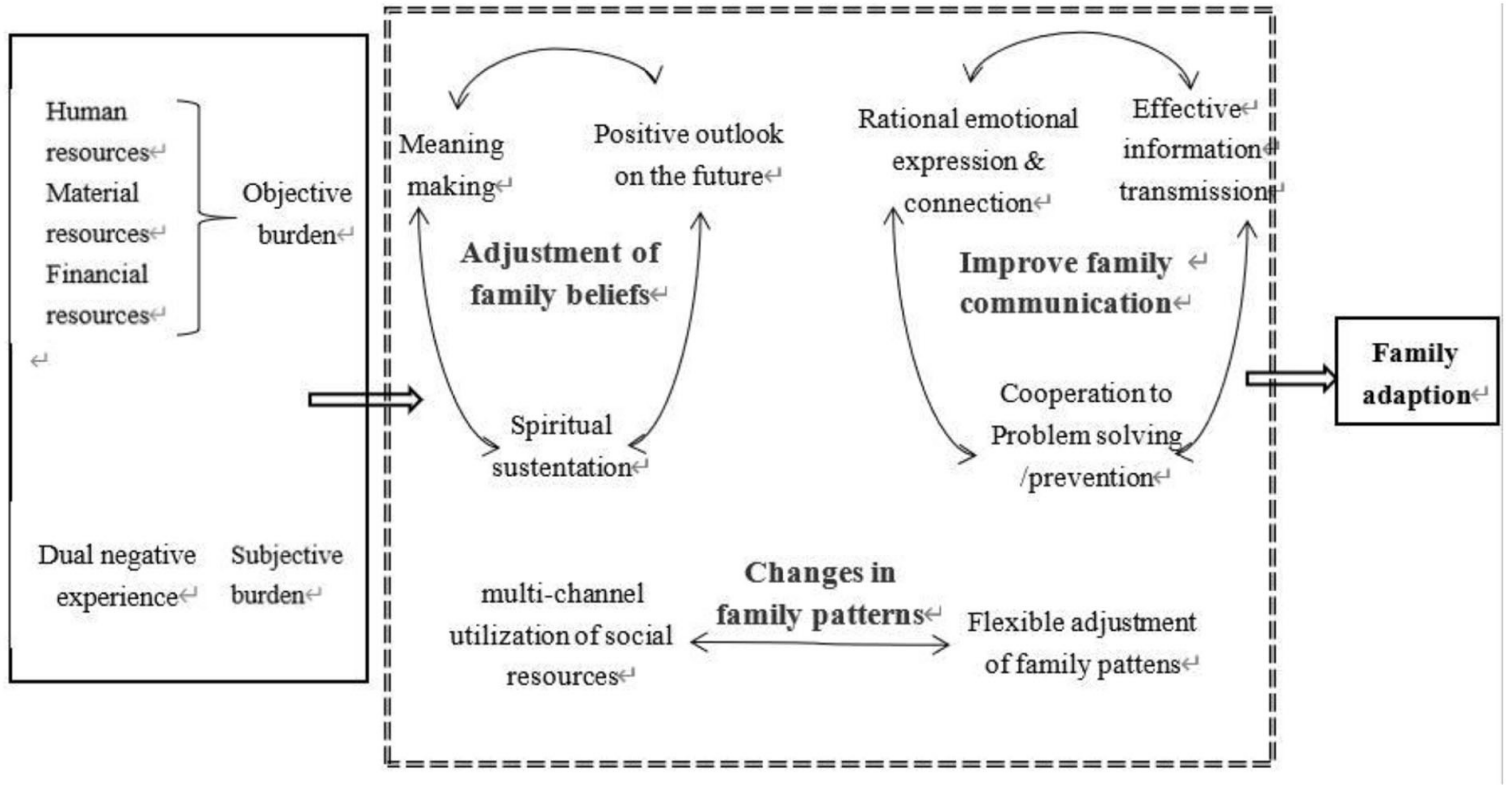
Dyadic cooperative coping and family resilience adjustment process.

The care of elderly individuals with disabilities at home is a long-term process that requires continuous support from family resources. The objective burden affects elderly individuals with disabilities and their caregivers synchronously, causing the elderly individuals with disabilities and their caregivers to have different experiences and leading to dyadic transfer. Although such families face a great deal of pressure, they are supported by the strength of positive coping. Table 2 summarizes the refined themes of this study, and Figure 1 illustrates these themes in the context of a logical and sequential model.

**Table 2:** Classification of theme and subtheme.

**Theme**	**Subtheme**	
Dyadic pressure	Heavy objective burden	
	Dual negative experience	Perceived low value in the elderly
		Low positive gaining in caregivers
	Dyadic interaction	
Dyadic cooperative coping	Adjustment of family beliefs	Giving meaning to stress
		Positive outlook on the future
		Spiritual sustentation
	Changes in family patterns	Flexible adjustment of family patterns
		Multi-channel utilization of social resources
	Improvement of family communication	Effective information transmission
		Rational emotional expression and emotional connection
		Cooperation to solve and prevent problems

### Theme 1: Dyadic pressure

The objective care burden of elderly individuals with disabilities increases with these individuals' degree of disability, and elderly individuals perceive themselves to have low value due to their disability. Caregivers receive low positive gains due to the stress of caring and other sources of pressure in life. Negative emotions are transmitted from one member of the dyad to the other and affect the functioning of the whole family.

#### Subtheme 1.1: Heavy objective care burden

Most caregivers clearly indicated that they faced different levels of care burden during the interview. Caregivers lack professional care ability and a professional care setting, and they face challenges such as advanced age, poor health, and lack of home care experience. Especially for those with low educational level, they are hard to find a channel to learn some basic care skills.

*It should be easier in the hospital. He has a 23* × *86 stone in his bladder, the prostate has calcified, and it's easy to get a urinary tract infection, so if there is a problem at home, how can I deal with it? (C3)*

Other respondents also reported unmet professional care needs, such as chronic disease complication management, disease control, recurrent episodes, and night care. The family lacks sufficient economic and manpower resources. For family living in rural community, there are few professional care centers to provide professional guide for them, while for family living in urban community, it often lacks a stable and reliable one to take care of the elderly at home. Many family members have to go out for working.

*No money to go to the hospital. The health care was not enough, and when my husband died, I was alone. My son just sent me to the hospital for a short period of treatment and then went back to home. (E5)*

Elderly people with disabilities have a high degree of dependence and lack social support, especially in rural community.

*Elderly people also need someone to accompany them. When there is no one to accompany him on the street, he can't the car bell, and thus, cannot even go outside from home for any social interaction. (C7)*

#### Subtheme 1.2: Dual negative experiences

##### The perceived low value of elderly individuals

The elderly respondents had highly negative experiences due to their disabilities, and the most prominent of these experiences were a reduced sense of self-efficacy, a diminished sense of worth, and a decreased sense of accomplishment. Some elderly respondents repeatedly used phrases such as “not good,” “useless,” and “It used to be okay.” When they require help from others for a long time and encounter the negative emotions of caregivers, elderly individuals with disabilities tend to have a variety of negative emotional experiences such as sensitivity, a sense of inferiority, pain and anxiety.

*I have to wait for others to bring me everything. Well, whether the food is hot or cold, it is good enough. That they cook it for me. Maybe they thought I was totally unprovoked when I sobbed. (E6)*

##### Low positive gains by caregivers

When elderly individuals with disabilities exhibit maladaptation to their role and poor compliance with care, caregivers tend to have negative psychological experiences, such as a low sense of reward.

*It's hard for you to persuade him. I told him to go out into the yard and get some sun. He was often forced to go, and he got angry. Leave him alone, and let him sleep as he likes. He has one foot in the grave already. (C7)*

Especially when caregivers do not receive positive emotional feedback from elderly individuals with disabilities, pain, disappointment, sadness, loneliness and other negative emotional experiences are more likely to occur. Several caregivers made statements such as the following: “there is nothing I can do,” “I'm just fed up with him,” and “she or he gets mad at me.”

When the resources required to care for elderly individuals with disabilities are insufficient, caregivers tend to have negative experiences such as impaired self-esteem or low self-esteem.

*I have also been trying to save money. People say they can't find a stingier person than me. I'm so choked up. (C8)*

#### Subtheme 1.3: Dyadic emotional transmission

The negative experiences of elderly individuals with disabilities and their caregivers can be transmitted easily to the rest of the family *via* language and behavior, thereby affecting the mood and mentality of other family members. When elderly individuals with disabilities suffer illness, the whole family environment becomes agitated.

*As long as there is a little discomfort, they will shout and keep making you unable to live peacefully. (C9)*

Even if verbal communication is limited, caregivers and elderly individuals can convey information *via* bodily expressions.

*He knows whether people dislike him or not by their facial expressions. (C7)*

When family members argue endlessly about caring for elderly individuals with disability, this situation can also aggravate the low value of elderly individuals with disabilities, leading them to wait passively for treatment.

*I should die so that others will not quarrel about caring for me. (E7)*

The negative attitudes of elderly individuals with disabilities also cause their caregivers to feel despair and anxiety.

*He just keeps saying such discouraging words. What can I do if I'm sad? Death is inevitable, sooner or later, the one who died in front was better, but the one who died in the back was more miserable. (C7)*

### Theme 2: Dyadic cooperative coping

Faced with objective care pressure and subjective negative experiences, elderly individuals with disabilities and their caregivers can nevertheless make positive adjustments to a certain extent and cooperate with their families to cope with difficulties. Ways of coping with such difficulties, including adjustments to family beliefs, changes in the family model, and improvements in the family communication process are shown in Figure 1.

#### Subtheme 2.1: Adjustments to family beliefs

A positive and effective family belief system can alleviate the negative experiences of caregivers and elderly individuals with disabilities in conditions of high stress and negative emotions, which is a prerequisite for beneficial adjustments. According to the interviews, caregivers and elderly people with disabilities were able to enhance the endogenous motivation of the family by actively exploring the positive significance of adversity, looking forward to the future and exhibiting faith.

##### Giving meaning to stress

Both caregivers and elderly individuals highlighted the responsibility and significance resulting from an inherited culture of “caring for the aged” and “feeding back to the parents.”

*They [the children] watch us take care of elderly individuals, and then they take care of us, and this is passed down from generation to generation; that is the reason for life. (C5)*

The old man thought that such care was a test for the child and a reward for his own efforts.

*After going home, I will depend on my son and daughter-in-law*.

*In life. They cannot leave me alone. (E5)*

Spouse caregivers embrace a spirit of “caring” and “sharing weal and woe” as well as the importance of the quality of being hardworking.

*I'm stronger than the others. As long as I work hard, life will be better. (C8)*

##### Positive outlook regarding the future

Both caregivers and elderly individuals noted the importance of moving forward in a positive manner. Both caregivers and elderly individuals were optimistic regarding the improvement of their disability status.

*As long as we take good care of her, we believe her situation will be better day by day. (C6)*

In addition, these dyads also create good plans and outlooks for their future life.

*We are in good health, and the children are working; he (the elderly person) should also think like us... We can live by growing my own food. (C8)*

##### Spiritual sustentation

When physical ailments interfere with daily life, elderly people with disabilities seek spiritual help by attending religious services.

*Worshiping Bodhisattva can make me feel better. I believe it can make me healthier and everything go well at home. (E4)*

Caregivers also take the initiative to confer spiritual meaning on caring behaviors, thereby stimulating endogenous motivation.

*My dad said to me, “Don't complain, no matter how hard it is, try to take good care of him. This kindness will be returned to your children. There will be smooth sailing and a good life in the future.” That made me feel even stronger. (C8)*

#### Subtheme 2.2: Changes in family patterns

After experiencing a disability event, the family model undergoes a dynamic adjustment process featuring changes in the needs of elderly individuals following the disability, and family and social resources become focused on caring for elderly individuals with disabilities to the greatest extent possible.

##### Flexible adjustments of family patterns

When the stress becomes excessive, elderly individuals with disabilities and caregivers adjust their cognitive stances. E1 took the initiative to let go of his dependence on his wife and transferred the responsibility for his sense of security to other family members: “it doesn't matter, I have my children to take care of me.” Under pressure from a lack of support from other family members, C5 took the initiative to rationalize her role: “Well, they are all busy. I am a daughter, and I must care more.”

In terms of practical actions, elderly individuals mainly focus on adhering to life and healthy self-management to reduce the burdens of care. On the other hand, caregivers attach importance to diseases suffered by elderly individuals, treat those diseases actively, and take the initiative to learn professional care knowledge and make preparations.

*I did my own laundry after I bought the wheelchair. (E6)*

*I feel I was careless before. I am preparing for the economy saving from now on. (C1)*

Mutual understanding and support among caregivers allows them to organize and distribute family resources as needed. Simultaneously, the connection between the nuclear family and the extended family is strengthened, and the extended family members become involved.

*We all take turns caring for each other. Basically, they are to be there during the day, and I come back at night. My aunt is also willing to help me, and my uncle is also taking care of me. (C1)*

##### Multichannel utilization of social resources

When the pressure becomes excessive, families of elderly people with disabilities engage in more interactions with the outside world. The timely acquisition of social resources can effectively solve problems and serve to moderate and reduce the emotional impact of family pressure on elderly people with disabilities at home. These social resources were generally easier to be obtained in urban community.

Families obtain information regarding diseases, nursing skills and medical resources from medical staff, information sources, relatives and friends.

*The doctor said that insulin should be taken in units of number. My son-in-law knew that a doctor had bought the medicine and the effect was good. (C7)*

Policies such as medical insurance, endowment insurance and material subsidies for persons with disabilities provide economic and material support for the families of elderly individuals with disabilities. Neighbors, friends and others also provide certain types of economic support and life assistance, which was more common in rural community.

*I can't get water, so I'll ask my neighbor next door to carry water. (E6)*

Targets of external emotional expression and dependence on the part of elderly individuals and caregivers mainly include the churches, community members, and family members of the patients.

*When I was crying next to the hospital bed, my elder sister said, “Why are you crying? Be strong; my family has also been in the hospital for half a year.” (C8)*

#### Subtheme 2.3: Improvement of family communication

An open and interactive mode of family communication not only enables family members to grasp the most effective information quickly and promotes flexible and efficient responses but also helps reduce the physiological and psychological pressure on the whole family.

##### Effective information transmission

Caregivers share information regarding the health status of elderly individuals with disabilities within the family, which not only improves the family's preparation for disease management and economic factors but also facilitates coordination among family members and reduces unnecessary misunderstandings.

*Elderly people can't cook; self-care is not possible. When I go home this time, I will discuss with them the possibility of taking turns to take care of the elderly individuals. (C5)*

##### Rational emotional expression and emotional connection

Elderly individuals with disabilities and their caregivers express their emotions appropriately and openly in various ways to reduce psychological pressure. One elderly man clearly expressed his gratitude to his caregivers for their efforts.

*I can't say that it is right to take care of me. I know my daughter loves me dearly, and I love her too. (E5)*

When one caregiver could no longer bear the elderly man's bad temper, he chose an appropriate time to express his feelings to the elderly man.

*I said to him: “At ordinary times, you still take it out on me when you are annoyed. Now, if you think about it, no matter how filial your children are, you will not be able to get up tomorrow morning.” (C7)*

In addition to dealing with emotions within the family, elderly individuals with disabilities and their caregivers also work together to cope with emotions caused by external factors. Following their disabilities, elderly individuals choose to be patient and not to complain when confronted by the gossip of others. The public opinion should be prevented from eroding the family structure and disrupting elderly people's stable condition.

*I have to recuperate and be patient with my illness. (E4)*

*I don't speak when he gets angry. If the house gets too noisy, people will certainly feel that the house is going to fall apart. (C8)*

Elderly individuals and their caregivers also employ empathy to understand each other's situations.

*Think of who will not be well; others also have it very hard. (E6)*

The combination of positive communication and cold treatment can help prevent a direct conflict.

*Everyone gets upset sometimes. (C1)*

*When she is sad, she tends to get angry. At that time, we usually try to persuade her first. (C6)*

A positive emotional connection and trust between elderly individuals with disabilities and their caregivers plays an important role in alleviating the negative emotions experienced by elderly individuals with disabilities and enhancing their sense of belonging and security.

*She talked me out of it. Called twice a day. I did feel better after the phone call. (E5)*

The gratitude, guilt and love expressed by elderly individuals enhance their caregivers' sense of achievement and self-significance.

*I just think that the elderly person will be well when she is well and that other pressures can be overcome. I just hope that she will be well, and there is no problem in going through hardships by myself. (C5)*

##### Cooperation to solve and prevent problems

Elderly individuals with disabilities and their caregivers communicate actively verbally or by other means to solve and prevent problems.

*I may not be familiar with him at the beginning, but gradually I will know something through his eyes and sleep. (C3)*

In regard to family affairs, caregivers and elderly individuals respect one another's right to make decisions.

*He does not block me from what I want to do, nor does he force me to do what I do not want to do, and I also let him be his own master. (C8)*

## Discussion

This study explored the dyadic life experiences of elderly individuals with disabilities and their caregivers from the perspective of family resilience. In general, three core processes in Walsh's family resilience model, such that family belief, family model and family communication, were supported in our finding. But as Walsh pointed out, three core processes can be organized and expressed in different ways, depending on family goals and preferences, structural configuration, adverse situations, and available resources (23). The findings of this study elucidate the most useful components of family functioning under the stress of caring for the disabled elderly in home setting, including the establishment of family belief systems, the ability to organize and co-operate family pattern, seek supporting, and proactive communication. By understanding the dyadic experiences of elderly individuals with disabilities and caregivers, the experience of dyadic cooperative coping with care stress in the context of home care for elderly individuals with disabilities in China has been deepened and enriched as showing in the intertwined subthemes, which are the original findings of this qualitative study.

Despite some common subthemes, cultural value influenced deeply in the dyadic experience. When looking at a family level, family culture is usually created under a unique background, involving social and political structure, economic state, natural factors and cultural tradition. Especially in cultural tradition, different from Western society, a “feedback model” was more prominent in Chinese society, which emphasizes on the intergenerational support (24). It undertakes to bear and educate offspring while also supporting parents. Relatives (especially children) who are related by blood are mainly responsible for providing care for the elderly (24, 25). As a traditional feedback model with profound cultural heritage, family care is still the first choice for the elderly, even if the family has gradually been unable to sustain the caregiver burden (26). In this context, the following elaboration on meaning making process, mutuality between the caregivers and the care recipients, and recommendations are easier to be understood.

### Dyadic negative experience

Long-term care for elderly individuals with disabilities leads to many care costs, such as high time and labor requirements, and caregivers bear tremendous physical, psychological and social burdens (21). In addition to objective pressure, elderly individuals with disabilities who receive home care are prone to feelings of inferiority, irritability and other negative emotions as a result of their disabilities due to disease, long-term disability and changes in their social role. If they have no effective way of communicating and talking, the initiative and enthusiasm of elderly individuals for self-care are greatly reduced (27). This study found that certain behaviors and reactions on the part of elderly individuals with disabilities, such as irritability, depression, or avoidance of seeing a doctor, convey to their caregivers the fact that these elderly individuals with disabilities are ignoring the management of their health and having negative experiences such as despair, and negative cognition. This situation causes caregivers to be vulnerable to negative cognitive and emotional infection (28), such that the caregivers feel helpless and believe that they are receiving few rewards, thus causing them to fail to meet or adapt to the needs of care as well as to exhibit fatigue, tiredness, or evasion. Caregivers who are responsible for the home-based care of elderly individuals with disabilities and who have negative experiences had much higher scores than did caregivers who exhibited obvious positive feelings (28). Similarly, caregivers' ineffective coping causes elderly individuals with disabilities to perceive that they are being told to give up, thus exacerbating the negative experiences of these elderly individuals. Therefore, caregivers' support and active coping are very important with respect to the task of directly alleviating the negative emotional experiences of elderly individuals with disabilities (29, 30).

### Dyadic cooperative coping

Elderly individuals with disabilities and their caregivers face common subjective and objective pressures. It is obvious that the relationship between elderly individuals and their caregivers should not merely pertain to caring and being cared for, in which context negative experience exert influence on this dyad. The family belief system helps family members confer a positive meaning on adversity and to re-evaluate and control their current situation, which is a core aspect of family resilience (16, 31, 32). According to this study, most elderly individuals with disabilities and their caregivers exhibit a sense of identity to “filial piety culture” and “caring for each other,” which to some extent alleviates elderly individuals with disabilities' feelings of anxiety and guilt and the caregivers' sense of low reward, thereby encouraging child caregivers and spouse caregivers to fulfill their obligations and responsibilities resulting from childhood support and marriage (33, 34). Family members' adherence to a common moral value system and conferral of positive meaning on difficulties can make the pressure encountered in the context of caring easier to accept (35). Against the social backdrop of urbanization and an aging population, family structure and concepts are constantly changing (36). Reasonable promotion and encouragement of “filial piety culture” is an important point of entry for the guidance of social atmosphere at the macro level. Simultaneously, religious beliefs endow families with spiritual sustenance, providing them with spiritual support and greatly enhancing their coping ability (37). Lietz et al. (38) confirmed that family confidence in the self-care ability of elderly individuals can improve their self-care ability and reduce the care burden of elderly individuals with disabilities. Therefore, the establishment of a stable and consistent family cognitive belief system is an important aspect of intervention.

### Family and social support recommended

Families have large social networks, and the more resources to which they have access, the stronger their resilience and the more easily they can adjust the family model and division of labor flexibly to suit the situation and allow the family to adapt to adversity (39). In the current situation of three-generation families in China, the supportive dual nurturing-feedback model dynamically shifts the focus of the family intergenerational relationship structure in a timely manner, which can help families cope with the dilemmas involved in care (39). Specifically, family resilience can be enhanced by changing roles within the family, altering lifestyles, adjusting needs and enhancing family care abilities (31, 40). Simultaneously, given a lack of internal resources in the family, family members actively seek external resources (40). For example, professional and reliable information provided by medical staff can guide family care methods and reduce family confusion and uncertainty (41). Community financial subsidies and care assistance can also alleviate some of the pressure of care. Simultaneously, as bio-psycho-social medical models have continued to develop, the importance of providing professional emotional support for elderly individuals and their caregivers has become increasingly prominent (42). Some studies from other countries have also shown that close emotional connection with the extended family is an important way of strengthening mutual support and overcoming emotional vulnerability (43). This study suggests that the community can provide a communication platform for patients with similar experiences and their families to obtain information, mutual emotional support, mutual guidance and encouragement as well as a sense of meaning from their efforts (32). When the community can provide long-term resources to the whole family to help the family better adjust its care model, even if the family itself has insufficient resources, larger social care network emerges to support family care. Therefore, the family itself, the expansion of the family and a beneficial community care model are crucial to family adaptation (28, 44).

### Effective family communication

Effective family communication involves clear information transmission, open emotional expression, wisdom and shared decision-making in the context of collaborative problem solving or prevention, and a process of joint efforts to achieve the same goal (45, 46). In line with the results of previous studies, a lack of information is a source of uncertainty for family members. Clear transmission of information can enable family members to understand their current situation fully and to optimize the care process, thereby helping them regain control and maintain family flexibility in the face of difficulties. A study have shown that increasing the frequency and depth of family members' mutual expression can enhance the family's adaptability (33). In this study, rational emotional expression on the part of family members was shown to alleviate negative experiences, which was consistent with previous study (47). Therefore, in terms of family information intervention, elderly individuals can be encouraged to communicate frankly with their family members to share their doubts, wishes and pains so that their families can gain a deeper understanding of the current situation and difficulties, mobilize their own enthusiasm in time and make effective adjustments (40).

### Limitations

This study has the following limitations. First, the data in this study are cross-sectional, which may not well reflect the changes in the dual care experience of the disabled elderly and their caregivers over time. Future study can consider the longitudinal design. Secondly, the inclusion criteria of the research subjects in this study did not focus on disability types of the elderly, and the exploration of specific family and disability types may be meaningful in future quantitative study. Finally, we focused primarily on the experience of the elderly and primary caregivers without involving other secondary family caregivers. Interaction within different family member with different roles may be considered and may give more in-depth findings.

## Conclusion

Though the sample was a little lean with only nine pairs, 18 participants with diverse socio-demographic characteristics were interviewed independently in this study which reached data saturation, and from the perspective of family resilience, this study explored the dyadic experiences of elderly individuals with disabilities and their caregivers in the context of home care. Both members of this dyad face physical, psychological, and social stress, which can be transmitted within the dyad. This study found that family resilience alleviates care stress and improves the negative experiences of elderly individuals with disabilities and their caregivers with respect to the aspects of family beliefs, family organization patterns and family communication patterns, which are closely related to the family's cultural background and community situation. The findings of this study elucidate the most useful components of family functioning under the stress of caring for the disabled elderly in home setting, including the establishment of family belief systems, the ability to organize and co-operate family pattern, seek supporting, and proactive communication. In the future, the dynamic process underlying longitudinal changes in family resilience and the care characteristics of different types of families or disabilities can be explored in further detail, and family resilience-based interventions can be developed in accordance with the core elements found by this study as a means of improving the resilience of the whole families of elderly individuals with disabilities at home. Other Asian countries in which older people receive similar forms of traditional, family-based, long-term care can also benefit from this study.

## Data availability statement

The raw data supporting the conclusions of this article will be made available by the authors, without undue reservation.

## Ethics statement

This study was approved by the Institutional Review Board of Research Institutions (IRB) in School of Nursing, Fudan University. The participants provided their written informed consent to participate in this study.

## Author contributions

All authors listed meet the authorship criteria according to the latest guidelines of the International Committee of Medical Journal Editors and all authors were in agreement with the manuscript. All authors had full access to all the data in the study, have been involved in drafting and/or revising the article critically, and given final approval of the version to be published.

## Funding

The research project was funded by National Social Science Foundation (20CSH016), Shanghai Yangfan Project (20YF1401900), and the Discipline Development Fund of Fudan University (FNSYL202008).

## Conflict of interest

The authors declare that the research was conducted in the absence of any commercial or financial relationships that could be construed as a potential conflict of interest.

## Publisher's note

All claims expressed in this article are solely those of the authors and do not necessarily represent those of their affiliated organizations, or those of the publisher, the editors and the reviewers. Any product that may be evaluated in this article, or claim that may be made by its manufacturer, is not guaranteed or endorsed by the publisher.

## References

[1] Chen L Akishita M Kuzuya M Kozaki K Kojima T Toba K. Future perspective on active ageing from rapidly ageing Asian countries. Eur Geriatr Med. (2012) 1:S7–8. 10.1016/j.eurger.2012.07.384

[2] Wei YG Wang ZC Wang HW Li Y Jiang ZY. Predicting population age structures of China, India, and Vietnam by 2030 based on compositional data. PLoS ONE. (2019) 14:212772. 10.1371/journal.pone.0212772 30973941 PMC6459537

[3] Li Y Guo H Li J Gong S Yi X. Research progress on the social ecosystem of family caregivers of the disabled elderly in my country. Nurs Res. (2020) 34:1764–7.

[4] O'Young B Gosney J Ahn C. The concept and epidemiology of disability. Phys Med Rehabil Clin N Am. (2019) 30:697–707. 10.1016/j.pmr.2019.07.012 31563163

[5] Wei L Yi H Hai-Lu Z. Elderly people with disabilities in China. J Am Geriatr Soc. (2019) 67:858–9. 10.1111/jgs.15793 30720879

[6] Corti MC Guralnik JM Salive ME Sorkin JD. Serum albumin level and physical disability as predictors of mortality in older persons. J Am Med Assoc. (1994) 272:1036–42. 10.1001/jama.1994.03520130074036 8089886

[7] Lampinen P Heikkinen E. Reduced mobility and physical activity as predictors of depressive symptoms among community-dwelling older adults: an eight-year follow-up study. Aging Clin Exp Res. (2003) 15:205–11. 10.1007/BF03324501 14582683

[8] Knodel J Minh Duc N. Grandparents and grandchildren: care and support in Myanmar, Thailand and Vietnam. Ageing Soc. (2015) 35:1960–88. 10.1017/S0144686X14000786

[9] Qiu D Hu M Yu Y Tang B Xiao S. Acceptability of psychosocial interventions for dementia caregivers: a systematic review. BMC Psychiatry. (2019) 19:23. 10.1186/s12888-018-1976-4 30642300 PMC6332684

[10] Gaugler J James B Johnson T Reimer J Solis M Weuve J. 2022 Alzheimer's disease facts and figures. Alzheimers Dement. (2022) 18:700–89. 10.1002/alz.12638 35289055

[11] Teahan A Lafferty A McAuliffe E Phelan A O'Sullivan L O'Shea D. Resilience in family caregiving for people with dementia: a systematic review. Int J Geriatr Psychiatry. (2018) 33:1582–95. 10.1002/gps.4972 30230018

[12] Stansfeld J Stoner CR Wenborn J Vernooij-Dassen M Moniz-Cook E Orrell M. Positive psychology outcome measures for family caregivers of people living with dementia: a systematic review. Int Psychogeriatr. (2017) 29:1281–96. 10.1017/S1041610217000655 28511738

[13] Wang A Bai X Lou T Pang J Tang S. Mitigating distress and promoting positive aspects of caring in caregivers of children and adolescents with schizophrenia: mediation effects of resilience, hope, and social support. Int J Ment Health Nurs. (2020) 29:80–91. 10.1111/inm.12651 31917518

[14] Yu X Zhang J. Resilience: the psychological mechanism for recovery and growth during stress. Adv Psycholog Sci. (2005) 13:658–65. 10.3969/j.issn.1671-3710.2005.05.016

[15] Lee I Lee EO Kim HS Park YS Song M Park YH. Concept development of family resilience: a study of Korean families with a chronically ill child. J Clin Nurs. (2004) 13:636–45. 10.1111/j.1365-2702.2004.00845.x 15189417

[16] Walsh F. A family resilience framework: innovative practice applications. Fam Relat. (2002) 51:130–7. 10.1111/j.1741-3729.2002.00130.x

[17] Hua H Cao Y. Belief, communication, and connection: research on the generation of anti -inverse force of children's family. Soc Work. (2019) 110:28–40. 10.3969/j.issn.1672-4828.2019.03.003

[18] Rahmati M Khaledi B Salari N Bazrafshan MR Haydarian A. The effects of religious and spiritual interventions on the resilience of family members of patients in the ICU. Shiraz E Med J. (2017) 18:13007. 10.5812/semj.13007

[19] Tong A Sainsbury P Craig J. Consolidated criteria for reporting qualitative research (COREQ): a 32-item checklist for interviews and focus groups. Int J Qual Health Care. (2007) 19:349–57. 10.1093/intqhc/mzm042 17872937

[20] Katz S Ford AB Moskowitz RW Jackson BA Jaffe MW. Studies of illness in the aged: the index of ADL: a standardized measure of biological and psychosocial function. J Am Med Assoc. (1963) 185:914–9. 10.1001/jama.1963.03060120024016 14044222

[21] Guba EG. Criteria for assessing the trustworthiness of naturalistic inquiries. Educat Commun Technol J. (1981) 29:75–91. 10.1007/BF02766777

[22] Lincoln YS Guba EG. But is it rigorous? Trustworthiness and authenticity in naturalistic evaluation new directions for program evaluation. New Direct Progr Eval. (1986) 1986:73–84. 10.1002/ev.1427

[23] Walsh F. Applying a family resilience framework in training, practice, and research: mastering the art of the possible. Fam Process. (2016) 55:616–32. 10.1111/famp.12260 27921306

[24] Qin K. Research on the change of China's pension model from the perspective of social embeddedness. Res Fin Iss. (2017) 11:133–8. 10.3969/j.issn.1000-176X.2017.11.019

[25] Li H. A Comparative Study of Chinese and Western Family Cultures from the Perspective of Historical Materialism. (PhD thesis), Shanghai University of Finance and Economics, Shanghai, China. CNKI (2020).

[26] Zhang Y. Analysis of the Influence of Different Pension Models on the Well-being of the Elderly in Rural China. (Master thesis), Shan Dong University, Jinan, China. CNKI (2016).33148248

[27] Zhao J Zhang Y Yu Z Wang R Li H Du C. Research on home rehabilitation experience of the disabled elderly in a county in Henan Province. Nurs Res. (2019) 33:3063–6. 10.12102/j.issn.1009-6493.2019.17.036

[28] Xu W Qian C Ma Y Shao S Wang H Jin G. A study on the status quo and influencing factors of the care burden of family caregivers for the disabled elderly in urban areas of Beijing. Chin J Dis Control. (2014) 18:663–6.

[29] Li L. Influencing Factors and Predictive Model Construction of Stigma Among the Disabled Elderly With Chronic Diseases. (Master thesis), Shanxi University of Traditional Chinese Medicine, Taiyuan, China. CNKI (2020).

[30] Sun J Yang W Wang Y Pan J Guo M Wang C. An empirical study on the effect of intergenerational support on depression in the disabled elderly. Chin J Soc Med. (2021) 38:154–7. 10.3969/j.issn.1673-5625.2021.02.009

[31] Saltzman WR Pynoos RS Lester P Layne CM Beardslee WR. Enhancing family resilience through family narrative co-construction. Clin Child Fam Psychol Rev. (2013) 16:294–310. 10.1007/s10567-013-0142-2 23797387

[32] Wong P Liamputtong P Koch S Rawson H. Searching for meaning: a grounded theory of family resilience in adult ICU. J Clin Nurs. (2019) 28:781–91. 10.1111/jocn.14673 30207613

[33] Chen HY Boore JRP. Living with a relative who has a spinal cord injury: a grounded theory approach. J Clin Nurs. (2009) 18:174–82. 10.1111/j.1365-2702.2008.02355.x 19120747

[34] Wang X. Research on Long-Term Care of the Elderly From the Perspective of Chinese Filial Piety Culture. (Master thesis), Jilin University, Changchun, China. CNKI (2017).

[35] Li LW McLaughlin SJ. Caregiver confidence: does it predict changes in disability among elderly home care recipients? Gerontologist. (2012) 52:79–88. 10.1093/geront/gnr073 21856746 PMC3297017

[36] Li B. Research on the weakening mechanism of family pension function under the background of urbanization. J Qinghai Normal Univ. (2019) 41:31–7. 10.16229/j.cnki.issn1000-5102.2019.06.006

[37] Jones KF Dorsett P Simpson G Briggs L. Moving forward on the journey: spirituality and family resilience after spinal cord injury. Rehabil Psychol. (2018) 63:521–31. 10.1037/rep0000229 30024204

[38] Lietz CA Julien-Chinn FJ Geiger JM Piel MH. Cultivating resilience in families who foster: understanding how families cope and adapt over time. Fam Process. (2016) 55:660–72. 10.1111/famp.12239 27489227

[39] Yang B Feldman MW Li S. The status of family resilience: effects of sustainable livelihoods in rural China. Soc Indic Res. (2021) 153:1041–64. 10.1007/s11205-020-02518-1

[40] Zerbetto SR Galera SAF Ruiz BO. Family resilience and chemical dependency: perception of mental health professionals. Rev Bras Enferm. (2017) 70:1184–90. 10.1590/0034-7167-2016-0476 29160478

[41] Ellis L Gergen J Wohlgemuth L Nolan MT Aslakson R. Empowering the “cheerers”: role of surgical intensive care unit nurses in enhancing family resilience. Am J Crit Care. (2016) 25:39–45. 10.4037/ajcc2016926 26724292

[42] West C Buettner P Stewart L Foster K Usher K. Resilience in families with a member with chronic pain: a mixed methods study. J Clin Nurs. (2012) 21:3532–45. 10.1111/j.1365-2702.2012.04271.x 23020890

[43] Wong P Liamputtong P Koch S Rawson H. The impact of social support networks on family resilience in an australian intensive care unit: a constructivist grounded theory. J Nurs Scholar. (2019) 51:68–80. 10.1111/jnu.12443 30471184

[44] Reader SK Pantaleao A Keeler CN Ruppe NM Kazak AE Rash-Ellis DL. Family resilience from the perspective of caregivers of youth with sickle cell disease. J Pediatr Hematol Oncol. (2020) 42:100–6. 10.1097/MPH.0000000000001682 31815887 PMC8524760

[45] Deist M Greeff AP. Living with a parent with dementia: a family resilience study. Dement Int J Soc Res Practice. (2017) 16:126–41. 10.1177/1471301215621853 26659440

[46] Walsh F. Family resilience: a framework for clinical practice. Fam Process. (2003) 42:1–18. 10.1111/j.1545-5300.2003.00001.x 12698595

[47] Gauvin-Lepage J. Traumatic brain injury in adolescence and the family resilience process: a case study. Sage Open Nurs. (2019) 5:2377960819848231. 10.1177/2377960819848231 33415241 PMC7774431

